# Aberrant Cortical Activity in Multiple GCaMP6-Expressing Transgenic Mouse Lines

**DOI:** 10.1523/ENEURO.0207-17.2017

**Published:** 2017-09-06

**Authors:** Nicholas A. Steinmetz, Christina Buetfering, Jerome Lecoq, Christian R. Lee, Andrew J. Peters, Elina A. K. Jacobs, Philip Coen, Douglas R. Ollerenshaw, Matthew T. Valley, Saskia E. J. de Vries, Marina Garrett, Jun Zhuang, Peter A. Groblewski, Sahar Manavi, Jesse Miles, Casey White, Eric Lee, Fiona Griffin, Joshua D. Larkin, Kate Roll, Sissy Cross, Thuyanh V. Nguyen, Rachael Larsen, Julie Pendergraft, Tanya Daigle, Bosiljka Tasic, Carol L. Thompson, Jack Waters, Shawn Olsen, David J. Margolis, Hongkui Zeng, Michael Hausser, Matteo Carandini, Kenneth D. Harris

**Affiliations:** 1 UCL Institute of Neurology, University College London, London, UK; 2 UCL Institute of Ophthalmology, University College London, London, UK; 3 Department of Neuroscience, Physiology, and Pharmacology, University College London, London, UK; 4 Wolfson Institute for Biomedical Research, University College London, London, UK; 5 Allen Institute for Brain Science, Seattle, WA; 6 Department of Cell Biology and Neuroscience, Rutgers, the State University of New Jersey, Piscataway, NJ

**Keywords:** Cortex, epilepsy, GCaMP, transgenic

## Abstract

Transgenic mouse lines are invaluable tools for neuroscience but, as with any technique, care must be taken to ensure that the tool itself does not unduly affect the system under study. Here we report aberrant electrical activity, similar to interictal spikes, and accompanying fluorescence events in some genotypes of transgenic mice expressing GCaMP6 genetically encoded calcium sensors. These epileptiform events have been observed particularly, but not exclusively, in mice with Emx1-Cre and Ai93 transgenes, of either sex, across multiple laboratories. The events occur at >0.1 Hz, are very large in amplitude (>1.0 mV local field potentials, >10% df/f widefield imaging signals), and typically cover large regions of cortex. Many properties of neuronal responses and behavior seem normal despite these events, although rare subjects exhibit overt generalized seizures. The underlying mechanisms of this phenomenon remain unclear, but we speculate about possible causes on the basis of diverse observations. We encourage researchers to be aware of these activity patterns while interpreting neuronal recordings from affected mouse lines and when considering which lines to study.

## Significance Statement

Genetically encoded calcium sensors have revolutionized neuroscience by providing a powerful way to measure neural activity with optical imaging. Transgenic mice that express these indicators have proved a particularly useful technology for their stability, ease of use, and breadth of expression. However, here we report that some of these mouse lines have major abnormalities in their brain activity, including large, broad events resembling epileptic activity. We confirmed these abnormalities across multiple laboratories and we provide methods to detect them. This finding is an important point of caution for researchers using genetically encoded calcium indicators to study neural activity.

## Introduction

A paramount challenge in neuroscience is the development of technologies that can measure neural activity across large regions of the brain with high temporal and spatial resolution. The refinement of calcium-sensitive fluorescent proteins, such as GCaMP6, has allowed for the minimally invasive measurement of neuronal activity with high signal-to-noise, at single neuron resolution, and on increasingly large spatial scales (Chen et al., 2013; Kim et al., 2013; Sofroniew et al., 2016; Stirman et al., 2016). Genetically encoded calcium-sensitive fluorescent proteins offer a number of advantages over viral expression or bulk injection: the experimental procedures can be less invasive; expression levels are more consistent among cells and across the subject’s lifetime; and they allow for imaging across wider regions of the brain. Accordingly, they have become popular tools, and several versions of this technology have been developed, in which different variations of the sensor protein are expressed under various promoters (Dana et al., 2014; Madisen et al., 2015; Wekselblatt et al., 2016).

Here we compare the neural activity, measured electrophysiologically and with imaging, from a variety of transgenic mouse lines expressing these proteins and find that a subset of these lines exhibit aberrant activity patterns resembling interictal spikes. Because of this resemblance, discussed further below, we refer to these events as “epileptiform.” We describe the characteristics of this activity, its incidence across different mouse lines, and speculate about its possible causes.

## Materials and Methods

### Mouse lines

We use the following abbreviations for transgenic mouse lines (expression notes here are not intended as definitive statements of expression patterns, just as general summaries).

- Ai93 (B6;129S6-*Igs7^tm93.1(tetO-GCaMP6f)Hze^*/J, Jax #024103, RRID:IMSR_JAX: 024104)
  - *Expresses GCaMP6f with Cre and tTA conditionality*
- Ai94 (B6.Cg-*Igs7^tm94.1(tetO-GCaMP6s)Hze^*/J, Jax #024104, RRID:IMSR_JAX: 024104)
  - *Expresses GCaMP6s with Cre and tTA conditionality*
- Ai95 (B6;129S-*Gt(ROSA)26Sor^tm95.1(CAG-GCaMP6f)Hze^*/J, Jax #024105, RRID:IMSR_JAX: 024105)
  - *Expresses GCaMP6f with Cre conditionality*
- Ai96 (B6;129S6-*Gt(ROSA)26Sor^tm96(CAG-GCaMP6s)Hze^*/J, Jax #024106, RRID:IMSR_JAX: 024106)
  - *Expresses GCaMP6s with Cre conditionality*
- Ai32 (B6;129S-Gt(ROSA)26Sor^tm32(CAG-COP4^*^H134R/EYFP)Hze^/J, Jax #012569, RRID:IMSR_JAX: 012569)
  - *Expresses ChR2 with Cre conditionality*
- tetO-G6s (B6;DBA-Tg(tetO-GCaMP6s)2Niell/J, Jax #024742, RRID:IMSR_JAX: 024742)
  - *Expresses GCaMP6s with tTA conditionality*
- Snap25-G6s (B6.Cg-*Snap25^tm3.1Hze^*/J, Jax #025111, RRID:IMSR_JAX: 025111)
  - *Expresses GCaMP6s in excitatory neurons*
- Camk2a-tTA (B6.Cg-Tg(Camk2a-tTA)1Mmay/DboJ, Jax #007004, RRID:IMSR_JAX: 007004)
  - *Expresses tTA in excitatory neurons and glia*
- Rosa26-ZtTA (STOCK *Gt(ROSA)26Sor^tm5(ACTB-tTA)Luo^*/J, Jax #012266, RRID:IMSR_JAX: 012266)
  - *Expresses tTA pan-neuronally*
- Emx1-Cre (B6.129S2-*Emx1^tm1(cre)Krj^*/J, Jax #005628, RRID:IMSR_JAX: 005628)
  - *Expresses Cre in excitatory neurons*
- Emx1-Cre-Kess (B6;CBA-Tg(Emx1-cre/ERT2)1Kess/SshiJ, Jax #027784, RRID:IMSR_JAX: 027784)
  - *Expresses Cre in excitatory neurons*
- Rbp4-Cre_KL100 (here “Rbp4-Cre” - STOCK Tg(Rbp4-cre) KL100Gsat/Mmucd, MMRRC#031125, RRID:MMRRC_031125-UCD)
  - *Expresses Cre predominantly in isocortical layer 5 excitatory neurons*
- Rorb-IRES2-Cre (here “Rorb-Cre” - B6;129S-*Rorb^tm1.1(cre)Hze^*/J,Jax#023526, RRID:IMSR_JAX: 023526)
  - *Expresses Cre predominantly in isocortical layer 4 excitatory neurons*
- Slc17a7-IRES2-Cre (here “Slc17a7-Cre” - B6;129S-*Slc17a7^tm1.1(cre)Hze^*/J,Jax#023527, RRID:IMSR_JAX: 023527)
  - *Expresses Cre in excitatory neurons, following Vglut1 expression*
- Scnn1a-Tg3-Cre (here “Scnn1a-Cre” - B6;C3-Tg(Scnn1a-cre)3Aibs/J, Jax#009613, RRID:IMSR_JAX: 009613)
  - *Expresses Cre predominantly in isocortical layer 4 excitatory neurons*
- Cux2-CreERT2 (here “Cux2-Cre” - B6(Cg)-*Cux2^tm3.1(cre/ERT2)Mull^*/Mmmh, MMRRC# 032779, RRID:MMRRC_032779-MU)
  - *Expresses Cre predominantly in isocortical layer 2/3 and 4 excitatory neurons*
- Ntsr1-Cre_GN220 (here “Ntsr1-Cre” - B6.FVB(Cg)-Tg(Ntsr1-cre)GN220Gsat/Mmucd, MMRC#030648, RRID:MMRRC_030648-UCD)
  - *Expresses Cre predominantly in isocortical layer 6 excitatory neurons*
- GP4.3 (C57BL/6J-Tg(Thy1-GCaMP6s)GP4.3Dkim/J, Jax #024275, RRID:IMSR_JAX: 024275)
  - *Expresses GCaMP6s in a subset of excitatory neurons*
- Pvalb-Cre (B6;129P2-*Pvalb^tm1(cre)Arbr^*/J, Jax #008069, RRID:IMSR_JAX: 008069)
  - *Expresses Cre predominantly in fast-spiking inhibitory interneurons*

### Electrophysiology

Electrophysiological recordings were performed at the Carandini/Harris Laboratory (University College London) and were conducted according to the UK Animals Scientific Procedures Act (1986) and under personal and project licenses released by the Home Office following appropriate ethics review. To prepare for electrophysiological recordings, mice (male or female; 8–30 weeks of age; numbers and genotypes as given in Table 1) were anesthetized briefly (<1 h), and a small craniotomy was drilled over the site of interest. The craniotomy was covered with Kwik-Cast elastomer for protection, and the mouse was allowed to recover. After several hours’ recovery, or over the subsequent several days, mice were head-fixed in the experimental apparatus. The elastomer was removed, and saline-based agar was applied over the craniotomy. The agar was covered with silicon oil to prevent drying. Recordings were performed with custom multisite silicon electrode arrays, inserted through the agar and through the dura. Signals were referenced to an external Ag/AgCl wire placed in the agar near the craniotomy. This external reference was shorted to the amplifier ground. Local field potential (LFP) signals were lowpass-filtered in hardware with a single-pole filter at 300 Hz and recorded to disk at 2.5 kHz. Subjects were awake and standing passively on a stationary platform. During the analyzed periods, subjects either viewed a “sparse noise” visual stimulus (8°-width white squares on a black background, at random times and positions) or no visual stimulus. A single channel from those in cortex was selected manually to be representative for further analysis.

**Table 1.** Incidence of epileptiform events in electrophysiological recordings of local field potentials in cortex

Genotype	Incidence of events (mice)
Emx1-Cre;Camk2a-tTA;Ai93	4/4
Emx1-Cre;Camk2a-tTA;Ai94	1/1*^*a*^*
Slc17a7-Cre;Ai95	0/1
Camk2a-tTA;tetO-G6s	0/2
Snap25-G6s	0/1
Pvalb-Cre;Ai32	0/2
wildtype (C57BL/6J)	0/4

Each count represents one mouse recorded electrophysiologically.

*^a^*The Emx1-Cre;Camk2a-tTA;Ai94 mouse was selected for electrophysiology on the basis of epileptiform events observed in imaging.

### Widefield imaging

At the Carandini/Harris Laboratory, widefield imaging was performed through the intact skull with a clear skull cap implanted. The clear skull cap implantation followed the method of Guo et al. (2014) but with some modifications. In brief, the dorsal surface of the skull was cleared of skin and periosteum and prepared with a brief application of green activator (Super-Bond C&B, Sun Medical Co.). A 3D-printed light-isolation cone surrounding the frontal and parietal bones was attached to the skull with cyanoacrylate (VetBond; World Precision Instruments) and the gaps between the cone and the skull were filled with L-type radiopaque polymer (Super-Bond C&B). A thin layer of cyanoacrylate was applied to the skull inside the cone and allowed to dry. Thin layers of UV-curing optical glue (Norland Optical Adhesives #81, Norland Products) were applied inside the cone and cured until the exposed skull was covered. A headplate was attached to the skull over the interparietal bone with Super-Bond polymer, and more polymer was applied around the headplate and cone. Imaging was conducted at 35 Hz with ∼10-ms exposures and 2 × 2 binning using a PCO Edge 5.5 CMOS camera and a macroscope (Scimedia THT-FLSP) with 1.0× condenser lens (Leica 10450028) and 0.63× objective lens (Leica 10450027). Illumination was by 470-nm LED (Cairn OptoLED, P1110/002/000). Illumination light passed through an excitation filter (Semrock FF01-466/40-25), a dichroic (425 nm; Chroma T425lpxr), and 3-mm-core optical fiber (Cairn P135/015/003), then reflected off another dichroic (495 nm; Semrock FF495-Di03-50x70) to the brain. Emitted light passed through the second dichroic and emission filter [Edmunds 525/50-55 (86-963)] to the camera. Data were compressed by computing the singular value decomposition of the 3D image stack, and individual pixel traces were reconstructed for analysis from the top 500 singular values. During imaging, the right eyes of the mice were illuminated with an infrared LED (SLS-0208A, Mightex; driven with LEDD1B, Thorlabs) and recorded with a camera (DMK 23U618, The Imaging Source) fitted with a zoom lens (Thorlabs MVL7000) and infrared filter (FEL0750, Thorlabs; with adapters SM2A53, SM2A6, and SM1L03, Thorlabs).

At the Häusser Laboratory (University College London), all surgical procedures were conducted under a license from the UK Home Office in accordance with the Animal (Scientific Procedures) Act 1986. Emx1-Cre-Kess;Camk2a-tTA;Ai93 mice and Emx1-Cre-Kess;Camk2a-tTA;Ai94 mice, between 6 and 17 weeks old, were anaesthetized using Isoflurane (0.5%–1%). A metal headplate with a 5-mm circular imaging well was fixed to the skull overlying the somatosensory cortex with dental acrylic (Super-Bond C&B). A craniotomy was drilled above S1 (right hemisphere, 2 mm posterior and 3.5 mm lateral of bregma). A cranial window, composed of a 3-mm circular glass coverslip glued to a 2-mm square glass with UV-curable optical cement (NOR-61, Norland Optical Adhesive) was press-fitted into the craniotomy and sealed using Vetbond before fixing it with dental acrylic. After at least 1 wk and up to 7 mo later, widefield imaging was performed at 15 Hz using a 470-nm LED (Thorlabs, M470L3) to illuminate the area. All except for the first imaging session of cb71-96 were done using an ORCA-Flash 4.0 V3 (Hamamatsu) camera and a 4× objective (4× Nikon Plan Fluorite Imaging Objective, 0.13 NA, 17.2 mm WD). Excitation light passed through an aspheric condenser lens (Thorlabs, ACL2520U-DG15) and a filter (Chroma ET470/40) and was reflected into the light path by a 495-nm longpass dichroic (Semrock, FF495-Di03-25x36) to reach the brain. Emitted light passed through the same 495-nm longpass dichroic as well as a 749-nm shortpass dichroic (Semrock, FF749SDi01-25x36x3) and an emission filter (Chroma HQ525/50) before reaching the camera. Spontaneous activity of awake mice running freely on a treadmill was acquired for 2–4 min. Only the first imaging session of cb71-96 was done using a Manta G609 camera (Allied Vision) and a 50-mm f/2.0 Ci Series fixed focal length lens (Edmund optics). Calcium signal time series were obtained from the average pixel intensity.

At the Margolis Laboratory (Rutgers University), widefield imaging was performed through the intact skull that was rendered transparent using methods similar to those described previously (Lee and Margolis, 2016). All procedures were conducted with the approval of the Rutgers University Institutional Animal Care and Use Committee. Mice were anesthetized with isoflurane (3% induction and 1.5% maintenance) in 100% oxygen, placed on a thermostatically controlled heating blanket (FHC) at 36°C, and mounted in a stereotaxic frame (Stoelting). The scalp was sterilized with betadine scrub and infiltrated with bupivacaine (0.25%) before incision. The scalp was reflected and the underlying skull was lightly scraped to detach muscle and periosteum and irrigated with sterile 0.9% saline. The skull was made transparent by applying a light-curable bonding agent (iBond Total Etch, Heraeus Kulzer International) followed by a transparent dental composite (Tetric Evoflow, Ivoclar Vivadent). A custom aluminum headpost was cemented to the right side of the skull, and the transparent portion of the skull was surrounded by a raised border constructed using another dental composite (Charisma, Heraeus Kulzer International). Mice were given carprofen (5 mg/kg) postoperatively. After a recovery period of at least 1 wk, mice were acclimated to handling and head fixation for an additional week before imaging. Mice were housed on a reversed light cycle, and all handling and awake imaging took place during the dark phase of the cycle. For imaging, mice were head fixed, and the transparent skull was covered with glycerol and a glass coverslip. Imaging was conducted using a custom macroscope that allowed for simultaneous imaging of nearly the entire left hemisphere and medial portions of the right hemisphere. The cortex was illuminated with 460-nm LED (Aculed VHL) powered by a Prizmatix current controller (BLCC-2). Excitation light passed through a filter (479/40; Semrock FF01-479/40-25) and was reflected by a dichroic mirror (Linos DC-Blue G38 1323 036) through the objective lens (Navitar 25 mm/f0.95 lens, inverted). GCaMP6f fluorescence (535/40; Chroma D535/40m emission filter) was acquired with a MiCam Ultima CMOS camera (Brainvision) fitted with a 50 mm/f0.95 lens (Navitar). The resulting 100 × 100-pixel image corresponded to an imaging area of ∼6.5 × 6.5 mm. Spontaneous activity was acquired in 20.47-s recordings at 100 frames/s with 20 s between recordings. Sixteen videos were acquired in each session. Changes in GCaMP6f relative fluorescence were calculated by subtracting the baseline, defined as the average intensity per pixel in the first 30 or 49 images, from each frame in a video and then dividing each frame by the same baseline. Calcium signal time series were obtained from the average intensity in 5 × 5-pixel regions of interest.

At the Allen Institute for Brain Science, widefield imaging was performed either through the intact skull using a modification of the method from Silasi et al. (2016) or through a 5-mm-diameter chronically implanted cranial window centered over the left visual cortex (Andermann et al., 2010; Zhuang et al., 2017). For through-skull imaging, the skull was exposed and cleared of periosteum, and a #1.5 borosilicate coverslip (Electron Microscopy Sciences, #72204-01) was fixed to the skull surface by a layer of clear cement (C&B Metabond, Sun Medical Co.). A 3D-printed light shield was fixed around the coverslip using additional Metabond, and the outward-facing surfaces were coated with an opaque resin (Lang Dental Jetliquid, MediMark). Mice with chronically implanted windows received a 5-mm-diameter craniotomy over the left hemisphere, centered at 2.7 mm lateral and 1.3 mm anterior to lambda. The craniotomy was sealed with a stack of three #1 coverslips, attached to each other using optical adhesive (Norland) and to the skull with Metabond. The window provided optical access to the left visual cortex, the posterior aspect of somatosensory cortex and medial aspect of dorsal retrosplenial cortex. In both cases, a custom-manufactured titanium headpost was fixed posterior to the lightshield/coverslip and dorsal to the cerebellum using Metabond. All surgical procedures were performed under isoflurane anesthesia and were approved by the Allen Institute Animal Care and Use Committee. Image acquisition used a Hamamatsu Flash4.0 v2 sCMOS camera running at half resolution (1024 × 1024) with a 10-ms rolling exposure (100 Hz). Images were produced by a tandem-lens macroscope (Scimedia THT-FLSP) with 1.0× tube and objective lenses (Leica 10450028) for through skull imaging or a 1.0× tube lens paired to a 1.6× objective lens (Leica 10450029) for imaging through the chronically implanted window. Epifluorescence illumination used a 470-nm LED (Thorlabs M470L3) filtered (Semrock FF01-474/27-50) and reflected by a dichroic mirror (Semrock FF495-Di03-50 × 70) through the objective lens. Fluorescence emission passed through a filter (Semrock FF01-525/45-50) to the camera. Data were spatially downsampled 16× by averaging, and calcium traces were obtained by subtracting and then dividing each pixel by its mean over the whole time series. At all locations, mice were either male or female and were 7–30 weeks of age.

### Two-Photon imaging

The subject (female Emx1-Cre;Camk2a-tTA;Ai94, 16 weeks of age, Fig. 4) was implanted at the Carandini/Harris Laboratory with a 4-mm-diameter glass window over visual, retrosplenial, and somatosensory cortices. Two-photon imaging was performed using a standard resonant B-Scope microscope (Thorlabs) equipped with a Nikon 16×, 0.8-NA water-immersion objective and controlled by ScanImage 4.2. Excitation light was provided at a wavelength of 970–980 nm through a tunable Ti:Sapphire laser (Chameleon Ultra II, Coherent). A custom light-isolation cylinder was placed around the imaging objective. Imaging was conducted at 30 Hz with a 500-µm-wide field of view in superficial layers (depth ∼200 µm) of somatosensory cortex (a site previously determined to show epileptiform events in widefield imaging). To extract a trace for detection of epileptiform events, all pixels from each frame were averaged together across the entire field of view [in MATLAB: squeeze(mean(mean(imstack,1),2)), where imstack is a 3D matrix of size nXpixels × nYpixels × nFrames].

### Doxycycline treatment

Mice treated with doxycycline were administered doxycycline orally via drinking water. A solution of 5% sucrose and 2 mg/ml doxycycline in tap water was given as drinking water from birth until 7 weeks of age. Treated and untreated animals were imaged as described above (widefield imaging in the Häusser laboratory).

### Testing for Cre-mediated Lox-STOP-Lox recombination in the germline

Mouse DNA was purified from 25-mg tail snips using Macherey-Nagel NucleoSpin Tissue 96 protocol adapted for the QIAgen QIAcube HT platform, yielding DNA concentrations of 15–60 ng/µL as measured by SpectraMax UV Spectrophotometer. Genotyping PCR reaction was performed using primers MG-2333 (5′-gctcgtttagtgaaccgtca-3′), MG-1099 (5′-tagccatggtgctgaggggatct-3′), and MG-2054 (5′-acagatcccgacccatttgctgt-3′). Amplification was performed in a 50-µL reaction volume and consisting of 9.5 µL nuclease-free water, 25 µL Taq master mix, 4.5 µL of each primer at 10 µM working concentration, and 2 µL template, and with the following PCR program: 3 min at 95°C for initial denaturation, followed by 45 cycles of 30 s at 95°C, 30 s at 60°C, and 90 s at 72°C, and completed with 10 min at 72°C. PCR products were visualized by capillary electrophoresis using the Advanced Analytical Fragment Analyzer dsDNA 910 protocol. PCR product size of 119 bp corresponds to intact LSL cassette (STOP^+^), and PCR product size of 183 bp corresponds to LSL deletion. We observed low-level amplification of 183-bp product in some samples, likely because of Cre-mediated recombination in tail tissue. All germline LSL deletion events were observed as complete absence of the 119-bp band (STOPdel^+^).

### Data analysis

Traces were analyzed by finding peaks and computing two parameters from them: prominence and full-width at half-prominence. Prominence is defined as the height of the peak relative to the greater of the two minima between the peak and its surrounding two higher peaks (or beginning/end of trace if no higher peak). Peak detection and calculation of these two parameters can be accomplished with the “findpeaks” function in MATLAB:
[peakValues,peakLocations,widths,prominences] = findpeaks(signal, t)


## Results

### Epileptiform events in LFP recordings

**Figure 1.**
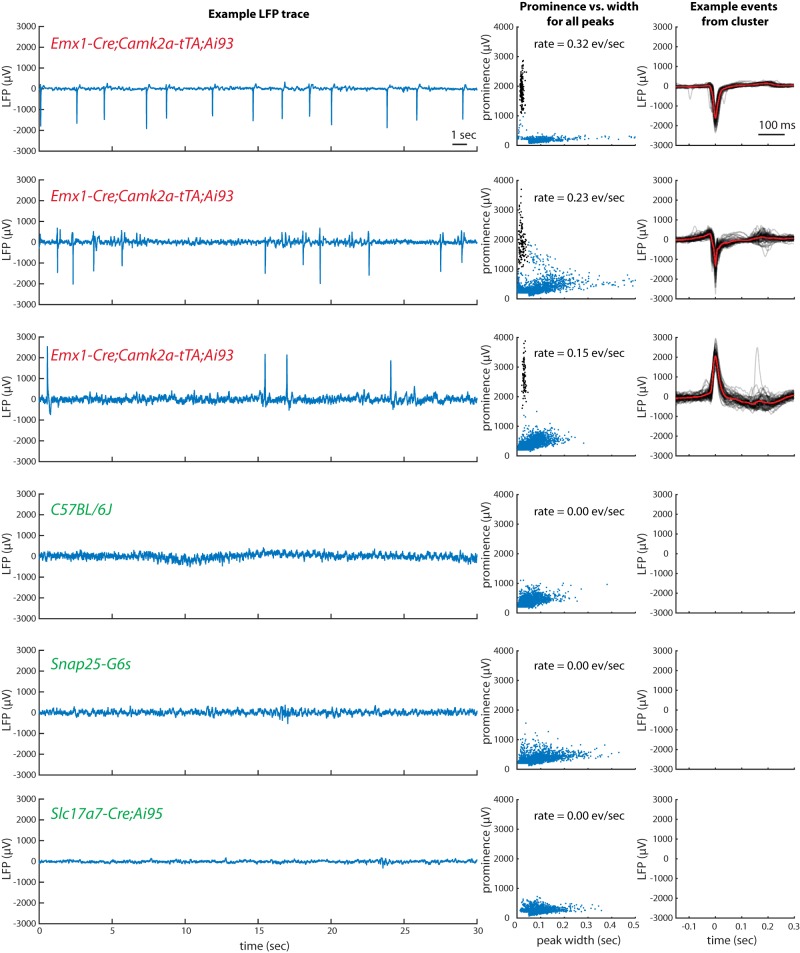
Epileptiform electrical activity in frontal cortex of some GCaMP6-expressing mice but not others. Left, each row contains an example segment of raw LFP data from each of six mice, with genotype identified in colored text. Middle, a plot of the prominence versus width of all peaks (see Methods) in the full LFP traces. Points highlighted in black were identified as a distinct cluster and included in the computation of event rate and the example events plotted at right. Right, 50 example events (gray) and the mean of all events (red). For the recording in row C, positive peaks rather than negative were analyzed.

We observed highly distinctive, aberrant electrophysiological events in LFP recordings from isocortex of some GCaMP-expressing lines. Epileptiform events were seen in all 4 recorded Emx1-Cre;Camk2a-tTA;Ai93 mice, but not in 10 mice from 5 other lines, including wild-type C57BL/6J (Fig. 1). Events were characterized by large amplitude, brief duration, stereotyped shape within a recording, and characteristic periodic spacing (“refractory” period of ∼0.5–1 s, overall rate ∼0.1–0.5 Hz). The presence of the events was visualized using a scatter plot of the prominence and width of all negative LFP peaks (see Methods). In recordings exhibiting these events, this plot revealed a distinct cluster of events of high prominence and short duration, outside the range of variation observed in other genotypes. Although the shapes of the events were stereotyped within a recording, they could vary significantly across recordings, presumably reflecting differences in the location of the recording site and reference relative to the origin and spatial spread of the event at a larger spatial scale (see also imaging results, below). These events were larger in amplitude than individual action potentials (>1 mV versus <750 µV), longer in duration (>10 ms versus <2 ms), and visible over a greater spatial range (>1 mm versus <100 µm), so they cannot reflect the activity of single neurons.

We tested for epileptiform events in electrophysiological recordings from 11 transgenic mice with six different combinations of transgenes, plus four wild-type C57BL/6J mice (Table 1). All tested Emx1-Cre;Camk2a-tTA;Ai93 mice (4/4) showed these events. We did not observe these events in mice of four other transgenic lines (Slc17a7-Cre;Ai95, Camk2a-tTA;tetO-G6s, Snap25-G6s, and Pvalb-Cre;Ai32) or in the wild-type mice. A single recorded Emx1-Cre;Camk2a-tTA;Ai94 mouse showed these events, but as this mouse was selected for this recording based on the presence of epileptiform events in imaging, their presence in electrophysiology in this mouse does not indicate that all mice of that genotype exhibit them.

### Epileptiform events in widefield imaging

**Figure 2.**
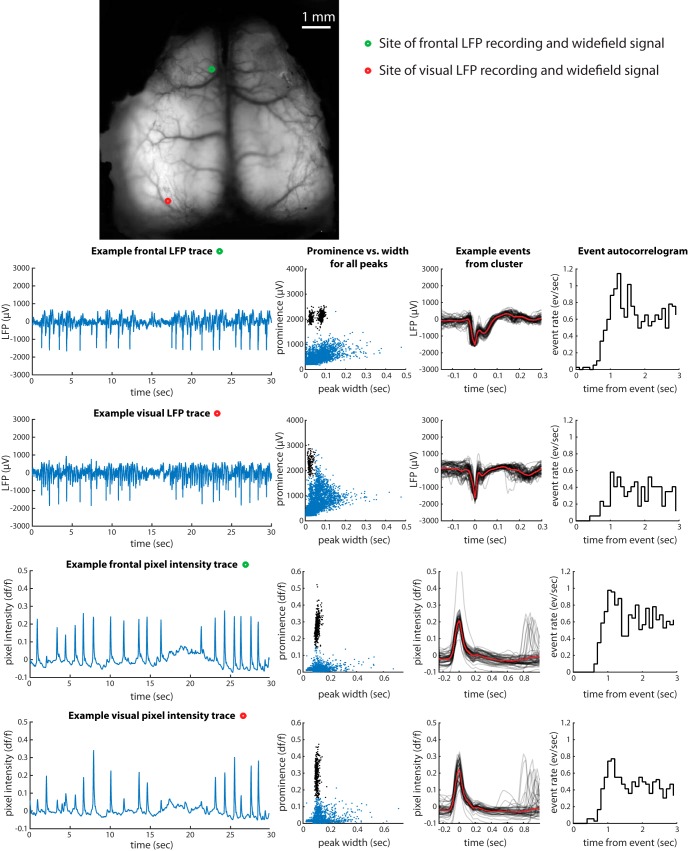
Comparison of events observed in LFP and widefield calcium imaging in the same mouse. Format as in Fig. 1. The two LFP recordings were made simultaneously with each other, but not simultaneously with the widefield imaging. In the frontal LFP recording, the two clusters in prominence versus width arise because of the double-peaked shape of the events in this mouse at this site: for some events, the width includes only the first peak; for some, it includes both.

Epileptiform events could also be unambiguously identified with widefield imaging using the same characteristics, i.e., large amplitude and brief duration relative to other events. In an example mouse, the rate of occurrence, refractory period, relative amplitude, and relative incidence across cortical regions were consistent, suggesting that the >1-mV events observed in LFP were the same events identified in imaging (Fig. 2). This was also true in other mice with nonsimultaneous measurements of LFP and imaging. We conclude that the large, brief “flashes” observed in imaging correspond to the large, brief LFP events.

**Figure 3.**
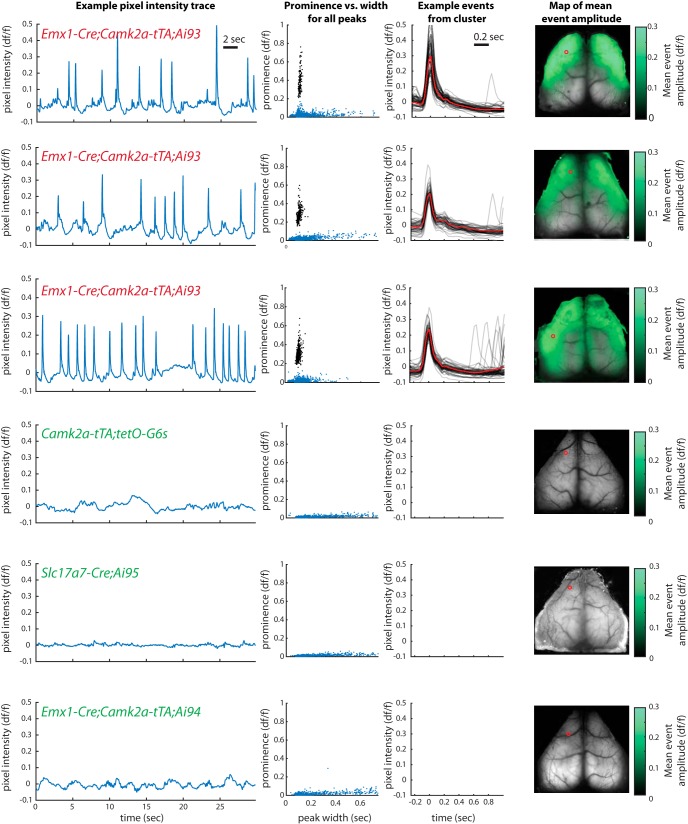
Incidence and spatial extent of epileptiform events observed in widefield calcium imaging. Format as in Fig. 1. The intensity trace and detected peaks come from the pixel indicated by the red circle on the brain image at right. Green coloration in the rightmost panel, overlaid on the mean image of the brain, represents the amplitude of the mean event at each point across the brain. See Videos 1, 2, and 3 for examples.

By observing the epileptiform events in widefield imaging, we measured the spatial extent of the events and characterized their incidence over a wider range of mice, including multiple genotypes and mice from multiple institutions. Widefield data from six more example mice are shown in Fig. 3 (see also Moveis 1, 2, and 3). The events were most frequently observed and relatively largest in an antero-lateral region of cortex including somatosensory, motor, and frontal cortex. Nevertheless, they could on some occasions be observed in visual cortex or retrosplenial cortex, usually with lower amplitude and/or relatively infrequent occurrence. They were nearly always bilaterally symmetric.

Movie 1. Emx1-Cre;Camk2a-tTA;Ai93 mouse. ***A***, The fluorescence signal imaged across the dorsal surface of the mouse brain (df/f). ***B***, at top, velocity of a rubber wheel under the forepaws of the mouse; below, traces of df/f over time from the four identified pixels (matching color points in the first panel). Red points indicate detected epileptiform events. ***C***, Two videos of the mouse. All movies play at half real time. Note that df/f scaling differs between movies for clarity of visualization. For further details of methodology, see Methods of widefield imaging at Carandini/Harris Laboratory. 10.1523/ENEURO.0207-17.2017.Movie.1

Movie 2. Emx1-Cre;Camk2a-tTA;Ai93 mouse. ***A***, The fluorescence signal imaged across the dorsal surface of the mouse brain (df/f). ***B***, at top, velocity of a rubber wheel under the forepaws of the mouse; below, traces of df/f over time from the four identified pixels (matching color points in the first panel). Red points indicate detected epileptiform events. ***C***, Two videos of the mouse. All movies play at half real time. Note that df/f scaling differs between movies for clarity of visualization. For further details of methodology, see Methods of widefield imaging at Carandini/Harris Laboratory. 10.1523/ENEURO.0207-17.2017.Movie.2

Movie 3. Slc17a7-Cre;Ai95 mouse. ***A***, The fluorescence signal imaged across the dorsal surface of the mouse brain (df/f). ***B***, at top, velocity of a rubber wheel under the forepaws of the mouse; below, traces of df/f over time from the four identified pixels (matching color points in the first panel). Red points indicate detected epileptiform events. ***C***, Two videos of the mouse. All movies play at half real time. Note that df/f scaling differs between movies for clarity of visualization. For further details of methodology, see Methods of widefield imaging at Carandini/Harris Laboratory. 10.1523/ENEURO.0207-17.2017.Movie.3

### Incidence

We observed epileptiform events in widefield imaging data from 63 of 175 mice across 17 genotypes and four labs (Table 2). These observations were made either using limited windows over restricted regions of cortex or, in separate mice, full-hemisphere windows. The incidence of events was greater in full-hemisphere recordings for matched genotypes. Considered together with the observations above that the epileptiform events often did not travel to visual cortex or had substantially lower amplitude there, we infer that they were often undetectable when limited regions of cortex were imaged alone.

**Table 2.** Incidence of epileptiform events as observed with widefield calcium imaging

Institute/laboratory	Cre genotype	tTA genotype	GCaMP genotype	Incidence of events in any size imaging window (mice)	Incidence of events in full-hemisphere imaging (mice)
**High incidence**					
Carandini/Harris, UCL	Emx1	Camk2a	Ai93	11/11	11/11
Allen Institute	Emx1	Camk2a	Ai93	9/18	5/7
Margolis, Rutgers	Emx1	Camk2a	Ai93	3/3	3/3
Margolis, Rutgers	Emx1	Rosa26	Ai93	7/7	7/7
Häusser, UCL	Emx1-Kess	Camk2a	Ai93	7/12	–
**Low incidence**					
Allen Institute	Slc17a7	Camk2a	Ai93	1/5	1/5
Allen Institute	Cux2	Camk2a	Ai93	1/7	–
Carandini/Harris, UCL	Emx1	Camk2a	Ai94	1/4	0/3
**No events observed**					
Allen Institute	Rbp4	Camk2a	Ai93	0/11	–
Allen Institute	Rorb	Camk2a	Ai93	0/3	–
Allen Institute	Scnn1a	Camk2a	Ai93	0/7	–
Häusser, UCL	Emx1-Kess	Camk2a*	Ai93	0/11	–
Carandini/Harris, UCL	Slc17a7		Ai95	0/7	0/7
Allen Institute	Emx1		Ai95	0/1	0/1
Allen Institute	Emx1		Ai96	0/3	0/3
Carandini/Harris, UCL		Camk2a	tetO-G6s	0/9	0/9
Allen Institute		Camk2a	tetO-G6s	0/6	–
Carandini/Harris, UCL			Snap25-G6s	0/4	0/4
Allen Institute			Snap25-G6s	0/2	–
Margolis, Rutgers			GP4.3	0/2	0/2

Epileptiform events were judged to occur by manually inspecting raw imaging videos, traces, and prominence-versus-width plots for peaks of the traces, as shown in Fig. 3. See Methods for details of mouse lines and imaging preparations. All mice in this table had either intact Cre conditionality or unknown conditionality. Camk2a* indicates that these mice were treated with doxycycline until 7 weeks (see below). See Table 2-1 for further details on these observations.

10.1523/ENEURO.0207-17.2017.1 Table 2-1 Details for all examined mice. The genotype for each transgene is indicated, alleles separated by a slash, with “wt” for wildtype. –, unknown information; NA, not applicable (e.g., for germline Cre-mediated recombination status of transgenes that cannot be subject to Cre-mediated recombination). Germline Cre recombination status is given as all alleles have intact LSL cassette (STOP^+^); all alleles have recombined LSL (STOPdel^+^); or one allele recombined and one intact (STOP^+^/STOPdel^+^; see Methods). Ages indicate the oldest age observed when no epileptiform events were reported or the youngest age at which epileptiform events were observed when they were. Download Table 2.1, XLS file.

**Figure 4.**
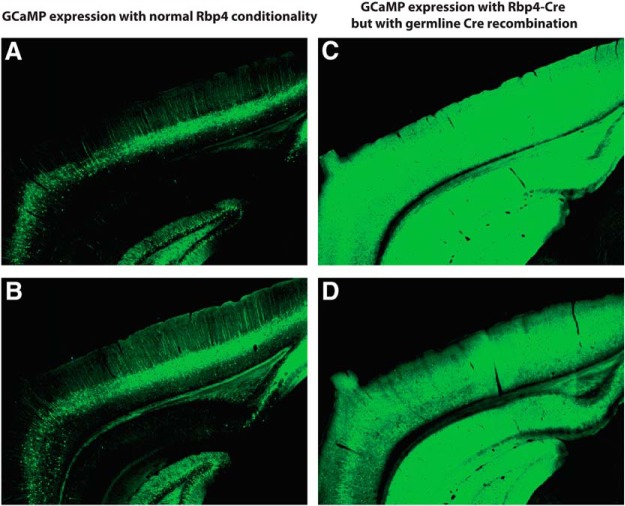
Germline Cre recombination results in widespread GCaMP expression. ***A***, ***B***, Native GCaMP6 fluorescence obtained using two-photon serial tomography from two Rbp4-Cre/wt;Camk2a-tTA/wt;Ai93 STOP^+^/Ai93 STOP^+^ mice, showing expression of GCaMP only in restricted populations of L5 cortical neurons and moderate expression in hippocampus. ***C***, ***D***, Similar images with matched intensity scale but from a Rbp4-Cre/wt;Camk2a-tTA/wt;Ai93 STOP^+^/Ai93 STOPdel mouse that had germline Cre recombination, showing high, widespread expression across cortex and hippocampus.

Epileptiform events were seen in the great majority of mice with the Emx1-Cre, Ai93, and either Camk2a-tTA or Rosa26-tTA transgenes (30/32 mice with full-hemisphere recordings; 46/75 in total). They were also seen in mice that expressed Ai93 with other drivers and in which germline Cre recombination had occurred (14/21 mice with limited-size windows, versus 0/14 in the same lines when germline Cre recombination did not occur; Table 3). Germline Cre recombination is the phenomenon in which ectopic expression of Cre in germline cells of previous generations results in deletion of the Lox-stop-Lox upstream of the reporter, and thus results in a loss of Cre conditionality in all cells of the animal (Schmidt-Supprian and Rajewsky, 2007). In these cases, the GCaMP expression pattern is determined by tTA expression alone, regardless of the cell types expressing Cre (Fig. 4; in all such cases, tTA expression was driven by the Camk2a promoter, resulting in broad and dense expression). In our experiments, we observed germline Cre recombination specifically with Emx1-Cre, Ntsr1-Cre, Rbp4-Cre, and Rorb-Cre. Although not all mice were tested for germline recombination, we observed a high probability of epileptiform events in Emx1-Cre;Camk2a-tTA;Ai93 mice with Cre conditionality intact (i.e., germline Cre recombination had been confirmed absent with genotype tests; 13/15 full-hemisphere recordings, 20/42 in total). We therefore conclude that mice with germline Cre recombination have a high probability of having epileptiform events for all tested Cre driver lines, but that the Emx1-Cre line is a special case in which epileptiform events were common regardless of germline recombination.

**Table 3.** Effects of germline Cre recombination on incidence of epileptiform events

Cre genotype	tTA genotype	GCaMP genotype	Incidence with germline Cre recombination	Incidence without germline Cre recombination
Emx1	Camk2a	Ai93	9/10	30/44
Ntsr1	Camk2a	Ai93	5/9	
Rbp4	Camk2a	Ai93	4/4	0/11
Rorb	Camk2a	Ai93	5/8	0/3

“With germline Cre recombination”, mice that had lost Cre conditionality; “without”, mice that were normal, i.e. expressed GCaMP only in subsets of neurons according to the Cre driver line used. In this table, all mice were imaged with widefield calcium imaging of any size window.

Only three mice with confirmed absence of germline recombination and genotype not including both Emx1-Cre and Ai93 were observed to have epileptiform events. Their genotypes were Cux2-Cre;Camk2a-tTA;Ai93 (1/7 mice recorded with limited-size windows), Emx1-Cre;Camk2a-tTA;Ai94 (1/4 mice overall, 0/3 with full-hemisphere imaging), and Slc17a7-Cre;Camk2a-tTA;Ai93 (1/5 mice recorded with full hemisphere windows). The other eight genotypes tested had zero observations of these events (0/53 mice).

### Detection of epileptiform events in two-photon imaging

**Figure 5.**
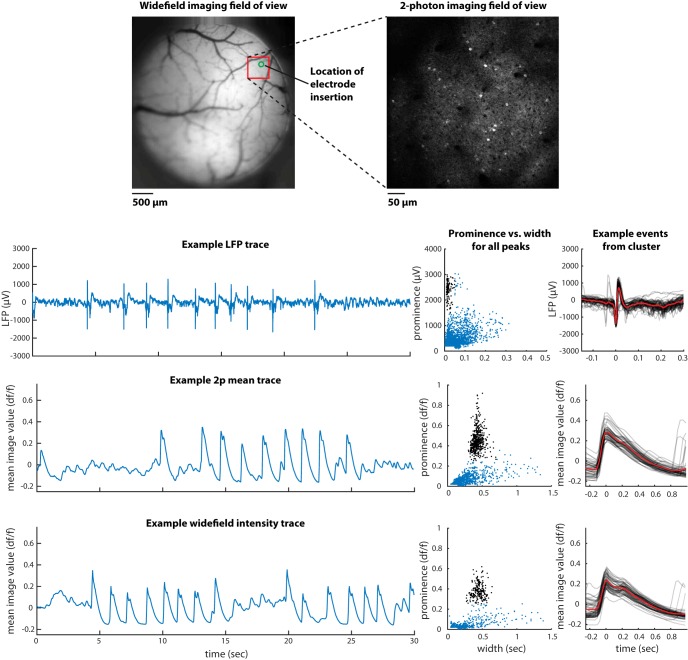
Epileptiform events observed in LFP, two-photon calcium imaging, and widefield calcium imaging in one individual mouse, but not simultaneously. Format as in Fig. 1. The genotype of the mouse was Emx1-Cre;Camk2a-tTA;Ai94 (expressing GCaMP6s). Two-photon trace was generated as the mean intensity of each frame across the entire field of view; widefield trace was generated as the mean within an ROI approximating the two-photon field of view.

The epileptiform events were also detectable in two-photon calcium imaging by creating a trace of the mean activity across the entire imaging field of view, including neurons and neuropil. In one mouse, we measured the neural activity nonsimultaneously in all three modalities (LFP, widefield, and two-photon) and found the same signatures in all cases (Fig. 5). We conclude that epileptiform events can be detected in at least some cases when present in the two-photon field of view. The caveat described above—that epileptiform events can be missed when limited fields of view are used that do not include the extent of the events—applies especially to two-photon imaging experiments with small fields of view, and for this reason we have not summarized in detail our observations with two-photon imaging.

### Generalized seizures

We observed generalized, tonic-clonic type seizures in 13 mice at three laboratories, including twice during a widefield imaging session. All of these were Emx1-Cre;Camk2a-tTA;Ai93 genotype or had germline Cre recombination with a different Cre line. These 13 mice comprise a small proportion of the total number of such mice that were studied (13/91, 14.3%), but this seizure incidence still represents a significant increase relative to other genotypes (0/92; *p* < 0.001, binomial test). However, mice were not monitored constantly in their home cages, so these numbers reflect a lower bound on the proportion of mice that had seizures.

### Prevention of events with doxycycline treatment

**Figure 6.**
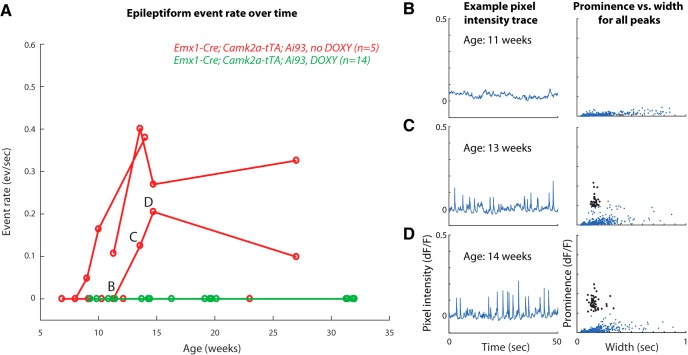
Epileptiform events fail to develop over time in mice treated with doxycycline until age 7 weeks. ***A***, Measured rates of epileptiform events in untreated (red) and doxycycline treated (green) mice by age at time of measurement. Connected points indicate observations from the same mouse. All doxycycline-treated mice failed to develop events over the measured time period. Note that the mice represented in this figure are a small subset of all measured mice, and we do not intend to claim that the time courses followed here are representative of all mice developing events. ***B***, ***C***, and ***D*** refer to the measurements corresponding to the figure panels at right. ***B–D***, Example traces and prominence versus width plots for an example untreated mouse showing development of events between 11 and 14 weeks.

To test the role of GCaMP6 expression during development, we treated a cohort of Emx1-Cre-Kess;Camk2a-tTA;Ai93 (*n* = 14) with doxycycline from birth until aged 7 weeks. Doxycycline blocks tTA activity and therefore prevented expression of GCaMP6 until removal from the drinking water. GCaMP6 expression levels were low shortly after removal of doxycycline and ramped up to levels comparable to untreated Emx1-Cre;Camk2a-tTA;Ai93 within 3–4 weeks. Using widefield imaging above S1, we found that all mice treated with doxycycline were free of epileptiform events in this brain area until the end of measurements (>30 weeks in 11 of 14; >11 weeks in the rest), although two mice did develop seizures despite lack of detected events. To compare this to the onset of epileptiform activity in untreated mice, we performed longitudinal widefield imaging in mice starting between ages 7 and 11 weeks (*n* = 5). In three of these untreated mice, epileptiform events were evident by age 13 weeks (Fig. 6), whereas two did not develop epileptiform events (measured until 23 weeks old). Altogether, although 60% of untreated mice (3/5) had epileptiform events by 13 weeks of age, no doxycycline-treated mouse (0/13) developed epileptiform events by this age or later. We conclude that doxycycline treatment until age 7 weeks eliminates the occurrence of epileptiform events.

## Discussion

Here we report the observation of aberrant neural activity resembling interictal spikes in some genotypes of transgenic mice expressing GCaMP6. This activity was most prominent in the Emx1-Cre;Camk2-tTA;Ai93 genotype, or in Camk2a-tTA;Ai93 mice that had undergone germline Cre recombination, but was also occasionally observed in other genotypes including one where GCaMP expression was driven by the Ai94 rather than Ai93 line.

These epileptiform events are strikingly distinct from activity observed in unaffected mice, in both amplitude and time course. Nevertheless, they can be easy to miss, for several reasons. First, there are no obvious behavioral manifestations, other than very rare generalized seizures. Second, the events are typically absent in visual cortex, a commonly studied area. Finally, the events may be obscured in two-photon imaging, even when present in the field of view, either because the sampling rate is slow relative to their duration or because they may primarily appear in neuropil and many individual neurons may show little relationship to them (e.g., as shown in Muldoon et al., 2015).

The presence of this aberrant activity complicates interpretation of studies on such mice. Certainly, when aberrant events are present in the recorded neuronal tissue, care must be taken when generalizing any conclusions on brain function drawn from these recordings to other mouse genotypes. For example, as the epileptiform events were substantially larger than other observed activity, they dominate measurements such as response variability and correlations across regions. Furthermore, our results suggest that the epileptiform events in these mice may frequently be undetectable in visual cortex although present in other brain regions. In such cases, it is possible that visual cortical activity shows more subtle alterations, not revealed by the present analyses.

Despite the large amplitude of these events, performance in learning and executing trained behavioral tasks was not immediately distinguishable between mice with and without them, and visual responses were similar to those in wild-type mice, when periods containing aberrant events were excluded (data not shown). Tonic-clonic seizures were also rare in these mice. Therefore, it is possible that some properties of cortical functional organization may still be measured with imaging in these mice, such as receptive field maps (Zhuang et al., 2017).

### Relationship to interictal spikes

In epileptic patients and animal models, interictal spikes are a commonly observed phenomenon. These large, brief electrical events are observable in electroencephalography, electrocorticography, and intracranial recordings and are considered to always be pathologic (Rodin et al., 2009). Whether they merely represent a symptom of a damaged brain or actively induce future seizures, or even act preventatively, remains disputed (Staley et al., 2011). Their exact characteristics vary by species and by variety of epilepsy, but in mice they have been defined as having <200-ms duration and large amplitude (generally >2× background activity amplitude, e.g., Erbayat-Altay et al., 2007). In these ways they are similar to the aberrant events we have observed, and we therefore referred to them as epileptiform. Furthermore, we observed a small proportion of mice to have generalized seizures among the mice with genotypes linked to a high incidence of epileptiform events. For these reasons, it seems possible or likely that the events we observed are similar or identical in origin to interictal spikes, and that the mice with these events may indeed be epileptic. To establish this more firmly, one would need 24-h observation of mice to establish seizure incidence, as well as a clearer understanding of the mechanisms underlying the events we observed. Note that although the epileptiform events observed here appear similar to interictal spikes, they are dissimilar to the spike-wave discharges noted in many rat strains and some mouse models of epilepsy, which occur at a much higher rate of 7–9 Hz (Wiest and Nicolelis, 2003; Fisher et al., 2014).

### Cause of aberrant activity

The cause of the epileptiform events is yet unknown and may result from a combination of effects, including Cre toxicity, tTA toxicity, and genetic background. Considering all observations together, however, the broad expression of GCaMP itself, particularly during development, seems likely to be a major factor.

The Cre enzyme is toxic (Schmidt-Supprian and Rajewsky, 2007), and Emx1-Cre specifically causes enhanced seizure susceptibility (Kim et al., 2013). Indeed we did not observe epileptiform events in any of the tested mice lacking Cre expression (i.e. Snap25-G6s, Camk2a-tTA;tetO-G6s, and wild-type C57BL/6J, *n* = 0/27 mice), consistent with a potential contributing role of Cre. However, regarding Emx1-Cre, we observed the events also in mice with Slc17a7-Cre, Ntsr1-Cre, Rbp4-Cre, and Rorb-Cre, so this particular Cre expression pattern cannot alone explain the effects.tTA can also be neurotoxic, causing hippocampal degeneration in mice of at least some strains, including 129 × 1/SvJ (related to the strain of origin for Ai93 and Ai94, 129S6/SvEvTac, which was not tested; Han et al., 2012). In addition to the interaction with tTA toxicity, strain itself might play a role, as different strains of mice have different seizure susceptibility, and the 129S3/SvImJ strain (again related but not identical to the strain of origin for Ai93 and Ai94) has higher seizure susceptibility than some other strains including C57BL/6J (Frankel et al., 2001). In general, the mice in this study were not congenic to a C57BL/6J background. However, the Rbp4-Cre mice with versus without germline Cre recombination have identical genetic backgrounds and identical levels of Cre and tTA, yet show large differences in event incidence (also true for Rorb-Cre). This comparison rules out a determining contribution of tTA toxicity or genetic background, though the possibility of an interaction remains.

GCaMP expression itself may play a role in the genesis of the epileptiform events. GCaMP binds calcium and thus buffers its intracellular concentration. Calcium plays many important roles in neurons, for example in synaptic transmission and in the expression of genes for synaptic plasticity, and disrupting these roles may accordingly alter network activity. Consistent with this, some genetic models of epilepsy in mice result from mutations to calcium channels (Letts et al., 1998). A major (if not the only) difference between the Rbp4-Cre;Camk2a-tTA;Ai93 mice with versus without germline Cre recombination is that the former will express GCaMP in a larger subset of neurons and possibly at higher levels than the latter, and the major difference in event incidence between these two groups of subjects (also true for Rorb-Cre) strongly suggests that the GCaMP expression itself plays a role.

Finally, we tested the role of GCaMP expression during development by suppressing tTA activity with doxycycline until 7 weeks postnatal in a cohort of mice. This treatment was effective in eliminating epileptiform events while preserving high expression levels in adulthood. Because expression of GCaMP in Ai93 depends on tTA activity, this manipulation should have prevented GCaMP expression during development, although doxycycline-treated mice expressed the same levels of Cre and shared the same genetic background as untreated mice. These experiments suggest that broad expression of GCaMP during development is a major contributing factor to epileptiform activity.

Taken together, these data suggest that a dominant role may be played by GCaMP6 expression itself, perhaps specifically during development. Cre toxicity, tTA toxicity, and genetic background may contribute, but appear unlikely to be determining factors. Whether the GCaMP expression is deleterious only when expressed in certain cell types or brain regions, whether it is deleterious only at certain restricted epochs in development, and whether different versions of the sensor have different severities of effects all remain unknown.

### Available alternative tools

For researchers wishing to pursue experiments with transgenic GCaMP6-expressing mice, some alternatives are available. Pan-excitatory expression with the GCaMP6f sensor can be accomplished with Ai93 using Slc17a7-Cre as an alternative to Emx1-Cre, or with Slc17a7-Cre;Ai95 (with lower expression levels). For the GCaMP6s sensor, the Camk2a-tTA;tetO-GCaMP6s mice (Wekselblatt et al., 2016) have expression levels comparable to Emx1-C re;Camk2a-tTA;Ai94 and have so far (*n* = 15 overall; *n* = 9 with full-hemisphere windows) not been observed to have epileptiform events in our hands.

### Notes of caution

Breeding strategies must take into account the possibility of germline Cre recombination, or else consistent and careful checks must be made to ensure that it has not occurred. It can generally be avoided by excluding females that are positive for both Cre and the *lox*P-flanked allele from breeding pairs. It can be tested for directly by genotyping or observation: e.g., in the present study, mice negative for Emx1-Cre but positive for Camk2a-tTA and Ai93 (littermates of those positive for all three) would still express GCaMP when germline Cre recombination has occurred.

Finally, though we have identified several lines with apparently normal electrophysiological activity, these mice nevertheless also express the same calcium-buffering proteins and at least one of Cre and tTA. Because a full, precise definition of “normal” and “abnormal” electrophysiological activity is unavailable, it is impossible to determine whether all activity in these mice is fully normal. We therefore suggest that confirming observations with multiple techniques—using both transgenic and wild-type mice—is an important experimental control whenever possible.

## Synthesis

Reviewing Editor: Thomas McHugh, RIKEN Brain Science Institute Decisions are customarily a result of the Reviewing Editor and the peer reviewers coming together and discussing their recommendations until a consensus is reached. When revisions are invited, a fact-based synthesis statement explaining their decision and outlining what is needed to prepare a revision will be listed below. The following reviewer(s) agreed to reveal their identity: Peyman Golshani.

Your manuscript has now been seen by two reviewers and we have discussed their comments to reach a consensus. Both reviewers find this to be an important study that should be published in a very timely fashion. Reviewer #1 synthesized the findings as “This is an excellent paper from multiple groups that shows that early developmental expression of GCAMP6 in the multiple Ai93, Ai94 X CamK2a TTA, Emx-Cre lines of mice causes interictal spikes. Emx-Cre driven expression seems to be most likely to cause discharges but other lines with early expression seem to also cause them. This will obviously cause problems as interictal discharges have been clearly shown to disrupt cognition and will complicate interpretation of experiments.”

While both reviewers are highly supportive of the work, they realize that this study may have immediate and important implications, likely leading to the culling of animals in many labs and complications of projects near completion. As such, we discussed several points for strengthening the findings and agreed the authors should address two main points when revising the study:

1) Both reviewers raised the possibility of including LFP data from the Thy1-GCAMP6 mice generated at Janelia (Dana et al, PLoS One 2014), to assess alternatives and extend the findings to an additional widely used line. If physiological ideally LFPs and/or calcium imaging from this line could be included that would be useful. If possible could the LFP and imaging work be done simultaneously with the Thy1 animals?

2) Additionally, both reviewers noted that LFP recordings were only conducted in one wild-type mouse. While the pathophysiology observed in the various transgenic lines would not be expected in wild-type recordings, the inclusion of a few more control animals would increase the rigor of the study.

Minor points:

1) Some important data are missing to have a better view of the situation. For example behavioral characterization or growth curves for the mice. Other important anatomical and physiological measurement as well (mortality, weight, cell count ...) may be available from the Allen Institute or JAX; if so, please add.

2) Could the authors speculate on the reasons for the difference between the Ai93 and Ai94 lines?
